# Glycoproteomic analysis of two mouse mammary cell lines during transforming growth factor (TGF)-β induced epithelial to mesenchymal transition

**DOI:** 10.1186/1477-5956-7-2

**Published:** 2009-01-08

**Authors:** Jennifer J Hill, Tammy-Lynn Tremblay, Christiane Cantin, Maureen O'Connor-McCourt, John F Kelly, Anne EG Lenferink

**Affiliations:** 1 Institute for Biological Sciences, National Research Council Canada, 100 Sussex Drive, Ottawa, ON K1A 0R6, Canada; 2 Biotechnology Research Institute, National Research Council Canada, 6100 Royalmount Ave., Montreal, QC H4P 2R2, Canada

## Abstract

**Background:**

TGF-β acts as an antiproliferative factor in normal epithelial cells and at early stages of oncogenesis. However, later in tumor development TGF-β can become tumor promoting through mechanisms including the induction of epithelial-to-mesenchymal transition (EMT), a process that is thought to contribute to tumor progression, invasion and metastasis. To identify EMT-related breast cancer therapeutic targets and biomarkers, we have used two proteomic approaches to find proteins that change in abundance upon the induction of EMT by TGF-β in two mouse mammary epithelial cell lines, NMuMG and BRI-JM01.

**Results:**

Preliminary experiments based on two-dimensional electrophoresis of a hydrophobic cell fraction identified only 5 differentially expressed proteins from BRI-JM01 cells. Since 3 of these proteins were glycoproteins, we next used the lectin, wheat germ agglutinin (WGA), to enrich for glycoproteins, followed by relative quantification of tryptic peptides using a label-free LC-MS based method. Using these approaches, we identified several proteins that are modulated during the EMT process, including cell adhesion molecules (several members of the Integrin family, Fibronectin, Activated leukocyte cell adhesion molecule, and Neural cell adhesion molecule 1) and regulators of cellular signaling (Tumor-associated calcium signal transducer 2, Basigin).

**Conclusion:**

Interestingly, despite the fact that TGF-β induces similar EMT phenotypes in NMuMG and BRI-JM01 cells, the proteomic results for the two cell lines showed only minimal overlap. These differences likely result in part from the conservative cut-off values used to define differentially-expressed proteins in these experiments. Alternatively, it is possible that the two cell lines may use different mechanisms to achieve an EMT transition.

## Background

Transforming growth factor beta (TGF-β) can act as both a tumor-suppressor and a tumor-promoter, depending on the cellular state and environment [1]. The tumor promoting role of TGF-β is linked to its ability to induce an epithelial-to-mesenchymal transition (EMT) in late stage cancers. EMT is characterized by a decrease in cell-cell adhesion, an increase in cell motility, and the activation of proteolysis, properties that are associated with tumor cell invasion and metastasis [2-5]. EMT also occurs throughout normal embryonic development and is critical for the formation of mesoderm during gastrulation [6]. However, due to the poorly regulated, stochastic nature of the EMT process during tumor development, cancer cells undergoing EMT often only use a subset of the molecular mechanisms utilized during EMT in embryogenesis [3].

The mouse mammary epithelial cell lines, BRI-JM01 and NMuMG, are independently derived cell lines that both undergo EMT upon exposure to TGF-β. NMuMG is a non-transformed cell line that is a well-established EMT model system, whereas the BRI-JM01 cell line was recently presented as an alternative for studying TGF-β-induced EMT [7,8]. In both cell lines, treatment with TGF-β induces similar changes in phenotype, including the loss or relocalization of epithelial markers (e.g. ZO-1 and E-cadherin), the rearrangement of actin and vimentin filaments, and an increase in motility.

Very few studies have explored the molecular mechanism of EMT in cancer cells by using proteomics to identify protein expression changes that are associated with this process [9,10]. Keshamouni et al. used iTRAQ to quantify protein expression changes in A549 lung cancer cells upon the induction of EMT by TGF-β [10]. Other proteomic studies on EMT have generally focused on a small number of selected proteins of interest [11,12].

Due to the wide dynamic range of protein expression, proteomic studies are generally limited to the analysis of the most abundant proteins in a complex mixture. Although cell surface and secreted proteins play an important role in mediating the invasive and metastatic properties of tumor cells, these proteins are often difficult to analyze by traditional proteomics methods such as two-dimensional gel electrophoresis (2DE), due to their hydrophobicity and relatively low abundance. Recently, inclusion of ASB-14 detergent has been reported to increase the number of hydrophobic proteins visible by 2DE, but this subset of proteins continues to be underrepresented in many proteomic studies.

Glycoproteins play an important role in the regulation and progression of many human diseases, including cancer [13,14]. Glycosylation is a post-translational modification that is particularly prevalent on secreted and membrane proteins, a subset of proteins that play an important role in the regulation of cell adhesion, motility, and EMT. Glycoproteins also make promising therapeutic and diagnostic targets for disease. In fact, most protein-based drugs on the market target glycoproteins, such as the blockbuster drugs Enbrel (TNF-α receptor-Fc fusion for rheumatoid arthritis/psoriasis) and Avastin (anti-VEGF monoclonal antibody for metastatic colon cancer). Glycoproteins also comprise the majority of clinical cancer biomarkers, including carcinoembryonic antigen (CEA), prostate-specific antigen (PSA), CA-125 antigen, and CA 15-3 antigen [15].

Since most cell surface and secreted proteins are glycosylated, it is possible to focus proteomic experiments on these proteins by enrichment using lectins, a family of proteins that bind specifically to glycans [16]. Many proteomic studies have exploited lectins for their ability to enrich glycoproteins and glycopeptides [17,18]. The majority of lectin studies are focused on the analysis of serum and plasma proteins in biomarker studies [17,19-21]. Other studies have highlighted the use of lectins with different glycan binding specificities as a means of simplifying complex protein samples before proteomic analysis, or to explore alterations in glycan structure that occur in many disease processes, including cancer [22,23]. These glycan variations may pin-point the disease stage and can be helpful for diagnosis [24,25].

To our knowledge, no proteomic studies have focused on glycoproteins in the context of EMT in a tumor cell model. Here, we have applied two proteomic analytical methods, 2DE and gel-free quantification of wheat germ agglutinin (WGA)-enriched fractions to identify proteins that change in abundance upon the induction of EMT. WGA is a well-characterized lectin that recognizes sialic acid and N-acetylglucosamine, a sugar present in the core structure of mammalian N-linked glycans. For comparison, protein changes were explored in both the BRI-JM01 and NMuMG cell lines.

## Methods

### Cell culture

BRI-JM01 cells were isolated, characterized and cultured as described [7]. Namru murine mammary gland (NMuMG) cells were obtained from ATCC and cultured as recommended. Human recombinant TGF-β1 (R&D Systems) was reconstituted according to the manufacturer's instructions. Cells were grown to 70% confluency in 150 mm dishes, after which TGF-β1 was added at a final concentration of 100 pM. After 24 hrs cells were washed 3 times with ice-cold Tris-buffered saline (TBS), collected in TBS using a rubber policeman, and pelleted by centrifugation (1500 rpm). Control (CTL) and TGF-β treated cells for each biological repetition were prepared in parallel. Cells for each of the three biological replications (BR1, BR2, and BR3) were prepared on three separate occasions, months apart.

### Two-dimensional electrophoresis

Cell pellets were lysed in 10 mM Tris-HCl, pH 7.4, 150 mM NaCl, and 1% Triton X-114 and hydrophobic proteins were enriched by phase separation at 30°C. Proteins in the detergent phase were acetone precipitated and resuspended in standard IEF buffer (7 M Urea, 2 M thiourea, 4% CHAPS, 1% DTT) with 1% ASB-14. Protein concentration was obtained by Bio-rad Protein Assay and 250 – 375 μg of protein was used to passively rehydrate IPG Strips pH 3–6 or 5–8 (Bio-rad) for 16–18 hrs. The strips were then submitted to isoelectric focusing (IEF) in the Protean IEF Cell (Bio-Rad). Prior to SDS-PAGE separation, proteins immobilized on the IPG strips were reduced (1% DTT) and alkylated (4% iodoacetamide) in SDS equilibration buffer (6 M urea, 30% glycerol, 2% SDS, 50 mM tris-HCl, pH 8.8). Separated proteins were visualized with Sypro Ruby for quantitative analysis followed by a silver nitrate staining for spot cutting. Gels were scanned with a Fluor-S imager (Bio-Rad) and analyzed using PDQuest (Bio-Rad). Protein ratios were calculated by averaging the ratio from all biological repeats in which the spot was quantified. Spots were selected for sequencing if they changed in intensity by ≥ 2 fold in at least 2 biological repetitions. Selected spots were excised manually, destained with a solution containing 15 mM potassium ferricyanide and 50 mM sodium thiosulfate, rinsed three times with water, and shrunk with acetonitrile. The gel pieces were swelled with 20 μl of trypsin solution (Promega, 0.01 μg/μl in 50 mM ammonium bicarbonate), and incubated overnight at 37°C.

### Enrichment of WGA binding proteins

Cell pellets were lysed by sonication in WGA binding buffer (WGA-BB: 50 mM Tris pH 6.5, 150 mM NaCl, 0.1 mM MnCl2, 0.5% Triton X-100) supplemented at 1:200 with a mammalian protease inhibitor cocktail (Sigma). Insoluble debris was pelleted by centrifugation at 11000 g for 20 minutes and the protein concentration of the cleared lysate was determined by the Bio-rad Protein Assay, using BSA as a standard. Lysates were adjusted to 0.75–1.0 mg/ml protein and 800 μg of protein was run through a spin column containing 150 μl packed volume of pre-washed WGA-conjugated agarose beads (4%, Sigma) 3 times by gravity and then saved as the 'flow-through' fraction. The beads were then washed 3 times with 500 μl WGA-BB, briefly spinning the column at 100 g for 1 minute after each wash. Bound glycoproteins were eluted with 4 × 125 μl of WGA elute buffer (50 mM Tris pH 6.5, 500 mM NaCl, 0.1 mM MnCl_2_, 0.5% Triton X-100, 0.3 M N-acetylglycosamine). Elution fractions were combined, precipitated with acetone, and resuspended in 50 mM Tris pH 7.4, 150 mM NaCl, 0.5% SDS, before being subjected to a tube-gel digest with trypsin, essentially as described [26]. Cysteines were reduced with 10 mM DTT and alkylated with 55 mM iodoacetamide. Peptides were extracted from the gel with 2 × 50 ul of 50% ACN/5% AcOH. Organic solvent was removed under vacuum before analysis by LC-MS(MS).

### SDS-PAGE and western blots

Proteins from whole cell lysates (input), WGA flow-through fraction (FT), and WGA elution (EL) fraction were separated by standard SDS-PAGE and visualized with ProQ-Emerald (Invitrogen) glycosylation specific stain, followed by Sypro Ruby total protein stain (Invitrogen). For western blots, SDS-PAGE separated proteins were transferred to nitrocellulose, blocked with 5% milk in Tris-buffered saline, and blotted by standard protocols with primary and HRP-labeled secondary antibodies. Proteins were visualized by fluorography with the ECL Plus Western Blotting Detection System (GE Healthcare). Primary antibodies used were anti-β-actin (clone AC-15, 1:5000 dilution) from Sigma-Aldrich; anti-Integrin-β4 (C-20, 1:200), anti-EMMPRIN (Basigin, B-5, 1:200), anti-Integrin-β2 (N-19, 1:200), anti-Integrin-β5 (H-104, 1:300 an H-96, 1:200), anti-Integrin-α3 (C-18, 1:200), anti-ALCAM (H-108, 1:200), anti-Tropomodulin-3 (C-13, 1:200), and anti-14-3-3σ (C-18, 1:200), all from Santa Cruz Biotechnology.

### Immunofluorescence microscopy

Cells were seeded in glass chamber slides (Nunc), grown to 70% confluency, and then maintained in the absence or presence of 100 pM TGF-β1 for 24 hrs. Cells were fixed with 4% para-formaldehyde in PBS (10 min, RT), permeabilized with 0.1% Triton X-100 (2 min, RT), and blocked with 10% FBS in PBS (30 min, RT). Cells were then incubated for 1 hr (RT) with a 1:100 dilution (in 10% FBS in PBS) of the following antibodies (all from Santa Cruz Biotechnology): anti-Clusterin (C-18, 1:100), anti-Integrin-α6 (H-87, 1:100), anti-Fibronectin (C-20, 1:100) and anti-NCAM (1.BB.495). After rinsing three times with PBS (RT) cells were incubated with either Alexa 488-conjugated donkey anti-goat IgGs, Alexa 488-conjugated donkey anti-rat IgG, or Alexa 488-conjugated donkey anti-rat IgG (all diluted 1:200, Invitrogen) for 30 minutes. Cells were then washed three times (PBS, RT), nuclei were counterstained with 4,6-diamidino-2-phenylindole (DAPI, 0.4 μg/mL), and then mounted using ProLong Gold anti-fade (Invitrogen). F-actin filaments were stained using rhodamine-labeled phalloidin (1:200) obtained from Invitrogen. Finally, fluorescent images were captured using a QImaging Retiga-2000R CCD camera mounted on a Leitz Aristoplan microscope. Images were processed using QCapture software (Meyer Instruments) and pseudo-colored using Photoshop CS3 (Adobe) software.

### Quantification by nano-LC-MS

For each BR, tryptic digests of the WGA elution fractions from CTL and TGF-β treated cells were analyzed 3 times by nano-LC-MS by subsequent, alternating injections on a Q-TOF Ultima coupled to a CapLC capillary LC system (Waters, Milford, MA). Samples were separated on a 5 cm × 75 μm BioBasic^® ^C18, 5 μm particle, PicoFrit^® ^column (New Objective, Inc., Woburn, MA) with a flow rate of ~250 nL/minute using a 42 minute gradient: 0–40% B (100% ACN/0.2% formic acid) over 37 minutes, 40–95% B over 5 minutes. Five minute washes in 50% B were followed by 20 minute blank gradients between each sample to minimize possible carryover effects. Continuum MS spectra were acquired every 2 seconds in the TOF-MS mode between m/z 400–2000. Under these conditions, the average peptide elutes over a time period of ~30–40 seconds, allowing 15–20 spectra to be collected per chromatographic peptide peak. Multiply charged ions were quantified and matched across different LC-MS runs by MatchRx [27], a software package developed in house that calculates the relative peptide abundance between subsequent LC-MS runs based on area under the chromatographic peak.

### Statistical analysis

For each biological repetition, individual MS runs were normalized so that the median peptide abundance of peptides matched and quantified in all MS runs is equal. Next, the following values were calculated in Microsoft Excel for every peptide quantified by MatchRx in ≥ 2 technical repetitions: the average and standard deviation of the peptide abundance in each of two samples (CTL and TGF-β) over the 3 triplicate LC-MS runs; log_2 _of the ratio of the abundance of the TGF-β sample relative to the abundance of the CTL sample (propagating the error from the standard deviations calculated above); the p value as calculated by a student's t-test comparing the 3 CTL abundance values to the 3 TGF-β abundance values. *Peptides *were designated as changed in abundance if they had a p value < 0.05 and a |log2ratio| > 0.75 (Filter A) or a p value < 0.05 and a |log2ratio| > 0.35 (Filter B).

*Proteins *were designated as differentially-expressed by grouping all peptides assigned to the protein and then calculating the probability of finding the number of 'changing' peptides, relative to the total number of peptides using the false positive rate calculated from the CTL1 versus CTL2 experiment (see text; 0.1 for Filter A and 0.25 for Filter B) and assuming a binomial distribution for a series of independent Bernoulli trials. Proteins with probabilities < 0.05 were considered to be changing in abundance, assuming it met all other criteria shown in our decision tree. Final protein ratios were calculated by summing the abundance of all peptides belonging to a given protein for each LC-MS repeat in a given biological repetition for both the CTL and TGF-β samples.

### Peptide identification by LC-MS/MS and database searching

Peptides were identified by LC-MS/MS on either a Q-TOF Ultima (Waters) or LTQ XL (Thermo) mass spectrometer. The Q-TOF Ultima was run using the LC setup described above with one of two instrument methods: (1) automatic switching to trigger MS/MS on the top 2 ions with a dynamic exclusion window of 40 seconds, or (2) inclusion list-MS/MS to specifically target differentially expressed peptide ions. Q-TOF data was searched against the NCBI nr database (04/05/2008; 6405498 entries) using Mascot with the following parameters (trypsin, 1 missed cleavage, peptide tolerance: 1.5 Da, MS/MS tolerance: 0.8 Da, modifications: carbamidomethyl (Cys, fixed), oxidized Met (variable). In auto-MS/MS files, only peptides with Mascot scores > 30 and a delta mass < 0.25 Da were considered. For inclusion list data, lower scores were considered after manual assessment. All Q-TOF spectra for differentially expressed peptides were manually verified. All 2DE spots were analyzed on the Q-TOF and required a minimum of 2 verified peptides to be identified. The LTQ XL mass spectrometer (Thermo) was coupled to a MDLC chromatography system (GE Healthcare). Samples were separated on a 5 cm × 75 μm BioBasic C18, 5 μm particle, PicoFrit column (New Objective) with a flow rate of ~300 nL/minute using a 30 minute gradient: 0–30% B over 14 minutes, 30–50% B over 14 minutes, 50–90% over 2 minutes. MS and MS/MS data were collected in enhanced profile and normal centroid mode, respectively. MS/MS was triggered by automatic switching on the top 2 peptides with a 40 second dynamic exclusion window and exclusion mass width of 0.1 (low) and 1.5 (high). CID settings were: isolation width = 1.0, normalized collision energy = 35, activation Q = 0.25, activation time = 30 ms. LTQ MSMS spectra were searched using BioworksBrowser 3.3.1 Build 7 against the ipi.mouse.v3.18 database (53788 entries, 06/23/2006) with the following parameters (trypsin; 2 missed cleavages; peptide tolerance = 2.0 Da; fragment tolerance = 1.0 Da; modifications: carbamidomethyl cysteine (fixed), oxidized methionine (variable)) and filtered to require X_corr _> 2.9 (2+), 3.4 (3+) and peptide probability < 1.0. A comparison of forward and reversed database search results from 10 LTQ auto-MS/MS files of WGA lectin fractions suggests a false positive identification rate of ~1% with these parameters (3 unique hits to reversed database versus 326 unique hits to forward database).

Due to the low redundancy and rich annotation detail, proteins in Table 1 and 2 were reassigned to mouse proteins from the Swiss-Prot database when an exact match for each peptide could be found using the pBLAST algorithm for short/nearly exact sequence [28,29]. All Swiss-Prot accession numbers from mouse and cow that contain the assigned peptide sequences are shown.

### MS/MS alignment with LC-MS data

Identifications from the MS/MS runs were aligned with the Q-TOF quantification nanoLC-MS data visually using MSight[30]. First, 6–10 peptides that were clearly identical were used as landmark alignments. Peptide identifications were imported onto the MS image produced from the MS/MS run and the equivalent peptide in the aligned nanoLC-MS quantification image was found manually. For a candidate ion in the quantification data to be assigned a MS/MS identification, the following criteria had to be met: (1) the charge state determined from Sequest or Mascot is identical to the candidate quantification peptide, (2) the theoretical monoisotopic m/z of the identified peptide and the monoisotopic m/z in the quantification Q-TOF data agree within 0.25 Da, (3) the candidate peptide is found within 2 minutes of its expected location based on the aligned images, (4) the relative positions of neighboring peptides are consistent between the identified peptide and the candidate quantification peptide, (5) no additional peptides fit the above description.

### Multiple Reaction Monitoring (selected ion monitoring)

Trypsin digested WGA elution fractions from a fourth biological repetition were analyzed on an LTQ using the chromatography conditions described above. For each injection, three MSMS daughter ions from each of two parent ions were monitored. Parent ions were subjected to CID (collision energy = 35%, Q = 0.25, time = 30 ms, isolation width = 3) and a limited range of m/z data, from -0.5 Da to +1.5 Da of the theoretical monoisotopic m/z of the daughter ion, was collected. Peptides were quantified by determining the area (total ion current) under the chromatographic peak for each transition in QualBrowser (Thermo), after baseline subtraction (polynomial order: 2; 10% below curve; tolerance: 0.01), smoothing (boxcar; 5 points), and peak identification (baseline window: 40; area noise factor: 3; peak noise factor: 10;). All analyses were performed in duplicate and peak areas for each transition were averaged. Peptide abundance ratios were calculated by dividing the peak area of each transition in the TGF-β sample by that from the CTL sample. Average ratios and standard deviations were calculated using all 3 transitions originating from the same parent ion.

## Results and discussion

### Phenotypic effects of TGF-β treatment on BRI-JM01 and NMuMG cells

**Figure 1:**
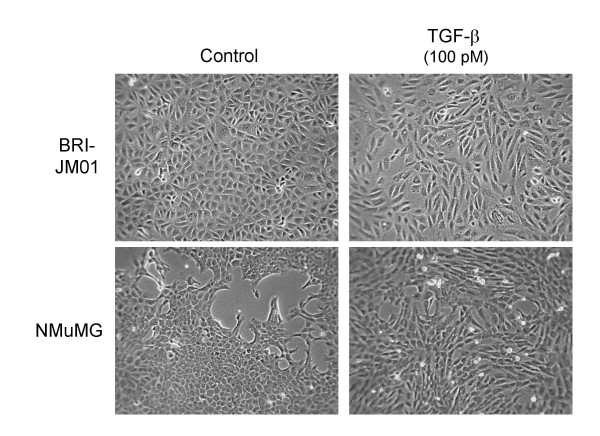
**BRI-JM01 and NMuMG cells undergo epithelial-to-mesenchymal transition upon treatment with 100 pM TGF-β1 for 24 hours**. After TGF-β1 treatment, both cell lines acquire a spindle-shape morphology, as shown by phase contrast microscopy.

Both the BRI-JM01 and NMuMG cells adopt a spindle-shape fibroblast-like morphology after 24 hrs in the presence of TGF-β1, as shown by phase contrast microscopy (Figure 1). This morphological change is accompanied by the down-regulation of various epithelial proteins (e.g. E-cadherin, tight junction protein ZO-1, and specific keratins), and the up-regulation of mesenchymal proteins (e.g. fibronectin, fibroblast-specific protein 1, α-smooth muscle actin and vimentin) [31]. The acquisition of this fibroblast-like phenotype correlates with increased cell motility in these cell lines [7,8], and in general allows epithelial cells to escape the structural limitations imposed by the existing tissue architecture. Motility and the ability to degrade the extracellular matrix are common features of invasive cells. Currently, the EMT process is thought to be directly linked to the invasive potential of a cancer cell.

### Two-dimensional gel electrophoresis studies: BRI-JM01 cells

**Figure 2:**
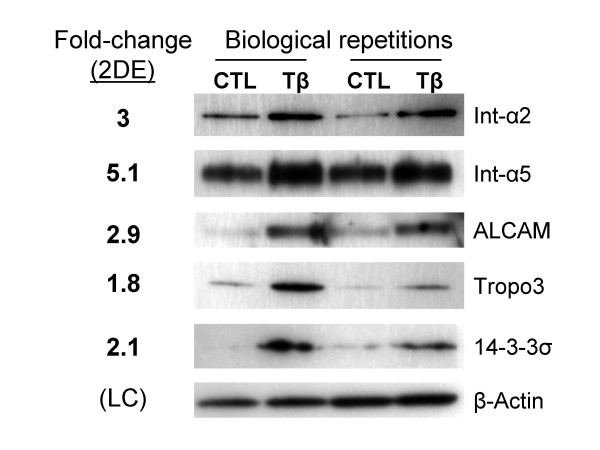
**Protein expression changes associated with TGF-β mediated EMT in BRI-JM01 cells, identified by 2DE**. 2DE studies identified 5 proteins that changed in expression after 24 hours of TGF-β treatment. Protein ratios (TGF-β/CTL) calculated from the 2DE data is shown in the left-hand column. All protein changes were subsequently verified by western blotting of BRI-JM01 cell lysates from 2 biological repeats, as shown. β-actin was used as a loading control (LC).

To identify EMT-related changes in protein expression in BRI-JM01 cells, our preliminary experiments utilized a traditional proteomics workflow: two dimensional gel electrophoresis followed by identification of differentially expressed protein spots by ESI-LC-MS/MS. Initial experiments using standard 2DE to separate BRI-JM01 whole cell lysates produced very complex 2-dimensional spot patterns that did not allow us to identify any consistent spot intensity changes after 24 hours of TGF-β treatment. Thus, we performed a phase separation in Triton X-114 to enrich for hydrophobic proteins and added ASB-14, a zwitterionic detergent, to the IEF buffer. We found that ASB-14 helped to solubilize high molecular weight transmembrane proteins and small membrane-associated proteins, such as the lipid-modified monomeric G-proteins (data not shown). Examples of the 2D-gels analyzed in these experiments are shown in Supplementary Figure 1 (see Additional File 1). Even after hydrophobic protein enrichment, only five proteins showed a consistent change in spot intensity after analysis of gels spanning two pH ranges (3–6 and 5–8) from three biological repetitions. All five differentially expressed proteins were up-regulated upon TGF-β treatment and included Integrin α2, Integrin α5, activated leukocyte cell adhesion molecule (ALCAM), Tropomodulin-3, and 14-3-3σ. All of the changes identified in this 2DE study were subsequently validated by western blotting of BRI-JM01 whole cell lysates, as shown in Figure 2. Of the five proteins identified, three are cell surface glycoproteins, suggesting that this subset of proteins may undergo more changes in protein expression than soluble, cytoplasmic proteins.

### Enrichment of glycoproteins from mouse breast epithelial cells undergoing EMT

**Figure 3:**
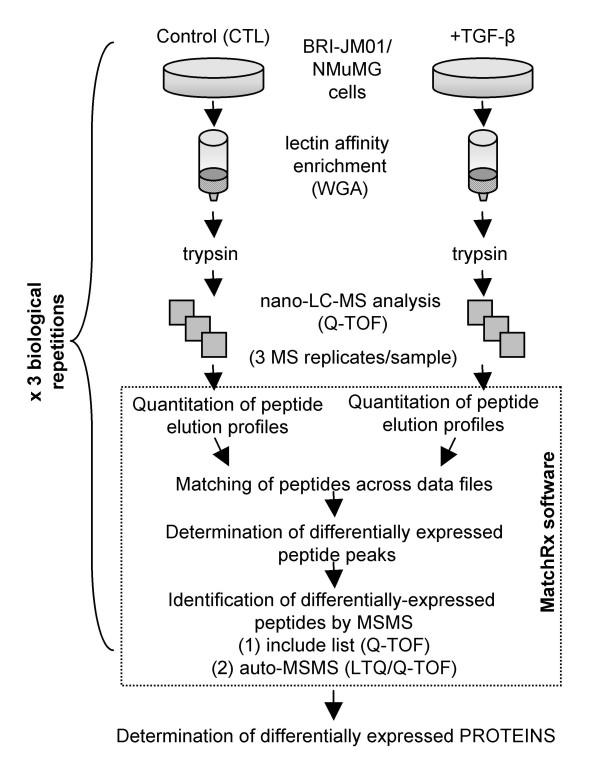
**Schematic of the lectin affinity workflow**. Immobilized wheat germ agglutinin was used to enrich glycosylated proteins from BRI-JM01 and NMuMg cells with or without the induction of EMT by TGF-β. Peptides in the WGA eluant were then quantified from nano-LC-MS data using a software package developed in-house, MatchRx, to identify differentially-expressed peptides.

Based on the results of our 2DE study, we chose to focus our analysis on glycoproteins, many of which are present on the cell surface or in the extracellular milieu. To do this, we enriched for glycoproteins using the lectin, wheat germ agglutinin (WGA). To eliminate the difficulties of separating hydrophobic membrane proteins by 2DE, we chose to quantify proteins by using a gel-free mass spectrometry-based method. We also expanded our experiments to include NMuMG cells to allow for comparison between two independently-derived cell lines that both undergo EMT in response to TGF-β. The workflow used in this analysis is summarized in Figure 3. Briefly, we compared peptide intensities in the WGA enriched fraction from both control and TGF-β treated cells (24 hour treatment) using a label-free approach to quantify individual peptide ions, i.e. the MS signal was integrated under the chromatographic peak in separate, subsequent LC-MS runs. Label-free quantification has many advantages over isotope labels, including minimal sample handling, the ability to compare multiple samples, increased protein coverage, and lower cost.

**Figure 4:**
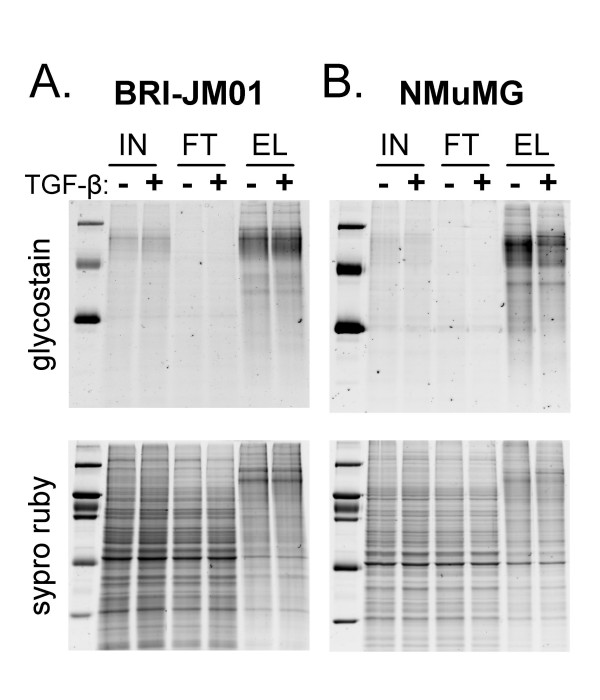
**Effective enrichment of glycoproteins by WGA lectin affinity workflow**. Immobilized wheat germ agglutinin was used to enrich glycosylated proteins from BRI-JM01 and NMuMG cells with or without the induction of EMT by TGF-β. Proteins from control and TGF-β treated BRI-JM01 (A) and NMuMG (B) cells were fractionated on a WGA affinity column. To determine the efficiency of glycoprotein enrichment by WGA, proteins from the unfractionated cellular lysate (input, 'IN'), the WGA flow-through ('FT') fraction, and WGA elution ('EL') fraction were separated by SDS-PAGE and visualized by both a glycoprotein-specific stain and Sypro Ruby, a total protein stain.

To confirm the efficient enrichment of glycoproteins from cell lysates of BRI-JM01 and NMuMG cells, we visualized proteins in the unfractionated cell lysate (input), WGA flow-through, and WGA elution fraction using a glycosylation-specific staining procedure (Figure 4). As expected, glycoproteins were depleted in the flow-through fractions and enriched in the elution fractions. Subsequent staining of the same gel with Sypro Ruby to visualize the total protein content of each fraction showed that each lane contained a similar amount of total protein. The overall protein banding pattern in the WGA eluant fraction was distinct from the banding pattern in the input and flow-through, confirming that a unique subpopulation of proteins is enriched by WGA. Interestingly, the majority of proteins appear to be unchanged in abundance after treatment with TGF-β.

Following digestion with trypsin, the WGA eluant fractions were analyzed in triplicate by LC-MS. A portion of the LC-MS data for each sample is shown in two-dimensional representation in Supplementary Figure 2 (see Additional File 1). The peptide maps from the control and TGF-β-treated samples derived from the same cell line displayed a high degree of similarity to each other, consistent with the gel protein staining patterns (Figure 4). However, the overall peptide elution patterns from the BRI-JM01 and NMuMG cells were quite different, indicating significant differences in WGA-binding glycoprotein content and abundance between these two cell lines.

### Quantitative analysis: statistical analysis of matched peptide sets

To estimate the quantitative error associated with the WGA workflow, we processed two cell pellets harvested from the same batch of control treated NMuMG cells (referred to as 'CTL-1' and 'CTL-2') in parallel with a TGF-β-treated sample. Theoretically, since CTL-1 and CTL-2 are derived from identically-prepared cells, all proteins should have the same abundance in both samples. Thus, any differences in peptide abundance presumably result from experimental error originating from the lectin affinity step or the LC-MS quantification. In contrast, abundance differences between the TGF-β-treated sample and the CTL samples would result from a combination of experimental error and true changes in protein abundance induced by the TGF-β treatment.

**Figure 5:**
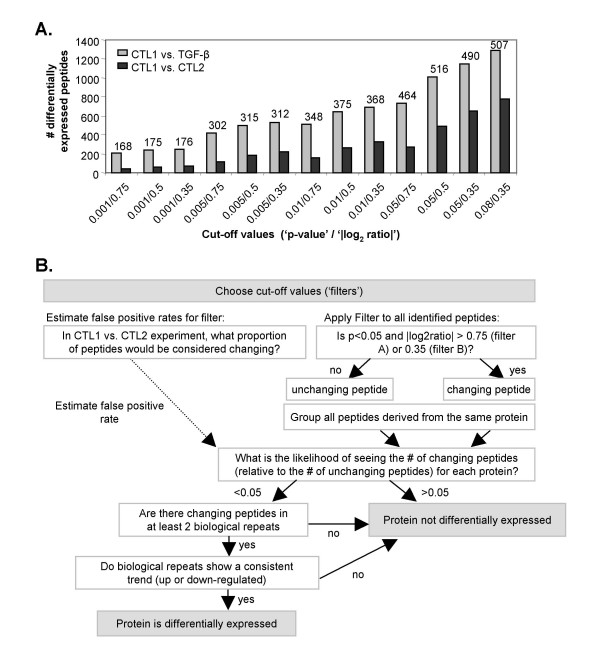
**Statistical analysis and decision-making tree used to identify protein expression changes in glycoprotein enriched fractions by a label-free LC-MS approach**. (A) Comparison of the number of differentially-expressed multiply-charged ions identified when different cut-off values for fold change (displayed as |log_2_(TGF-β/CTL)|) and p value are used (gray bars). As an estimate of the number of false positives identified with each set of cutoff values, two separately prepared control samples (CTL-1 and CTL-2) were analyzed in a similar fashion (black bars). The difference between the number of differentially-expressed ions identified in the TGF-β/CTL comparison and the CTL-2/CTL-1 comparison is shown above each set of bars and represents the number of real, truly differentially expressed ions. (B) The decision tree used to determine whether a given protein is differentially-expressed is shown. Briefly, all peptides that were identified in any given biological repeats were used to determine initial statistical significance. Subsequent filtering then required that differentially-expressed peptides were identified in at least 2 biological repeats and that the remaining biological repeat showed a consistent trend towards up- or down-regulation.

In this experiment, 2733 multiply-charged ions were matched and quantified in all three samples (CTL1, CTL2, and TGF-β). Of these, 273 ions were differentially-expressed between the CTL-1 and CTL-2 samples with a fold change greater than 68% (|log2 ratio| > 0.75) and a p value of < 0.05. With these same fold change and p-value cut-offs, 737 ions were differentially expressed between the CTL-1 and TGF-β samples. The results from the CTL-1/CTL-2 comparison, where no peptide ions are expected to be truly differentially expressed, suggest that 273 of the 737 differentially expressed ions identified in the TGF-β/CTL-1 comparison are probably false positives and, therefore, are not truly differentially expressed. On the other hand, 464 of these ions (737 minus 273) likely represent true changes in peptide abundance induced by TGF-β. Figure 5A shows the number of ions that pass 13 different criteria for differential expression, each criteria using a different set of fold change and p-value cut-offs. Importantly, the number of ions found to be differentially expressed between the CTL-1 and TGF-β sample is consistently higher than the number of ions found to be differentially expressed between CTL-1 and CTL-2, demonstrating that peptide expression changes induced by treatment with TGF-β can be detected. The estimated number of truly differentially expressed ions identified by each set of cut-off values is shown above the data bars in Figure 5A. In general, when stringent cut-off values are used, such as a lower p-value or a higher fold change, the estimated number of false positives is lower. However, more forgiving cut-off values identified a larger number of ions that are potentially differentially expressed in response to TGF-β treatment. This trade-off between sensitivity (the ability to detect differentially expressed peptides) and selectivity (minimizing false positive findings) is a common issue in proteomic studies.

To identify glycoproteins that are differentially expressed upon TGF-β treatment, WGA enriched fractions from three separate biological repetitions (BR1, BR2, and BR3) in both the BRI-JM01 and NMuMG cell lines were analyzed and the decision tree shown in Figure 5B was applied. To maximize our sensitivity, we applied two sets of cut-off values to identify differentially-expressed peptides. The use of 2 filters at the peptide level allowed for the identification of proteins that are more strongly regulated, but represented by only a few peptides (Filter A), as well as proteins that are more weakly regulated, but represented by larger number of peptides that lend statistical weight to the findings (Filter B). To minimize false positive findings, we took advantage of the additional information provided by quantification of multiple peptides from a single protein, as well as quantification of the same peptide in multiple biological repetitions. At the protein level, we required the presence of statistically significant differentially-expressed peptides in at least 2 biological repetitions, with the third biological repeat showing the same upward or downward trend (see Figure 5B).

### Identification and validation of WGA binding proteins that are differentially expressed after induction of EMT by TGF-β

Overall, 144 proteins were quantified from the WGA elution fraction of the BRI-JM01 cell line and 97 proteins were quantified from the NMuMG cell line, in a minimum of 2 biological repetitions. Application of the decision tree described above led to the identification of several proteins as being regulated by TGF-β treatment in BRI-JM01 and NMuMG cells, as shown in Table 1 and Table 2 respectively. Details on the individual peptides that were identified, including database searching scores and peptide-level quantitative information, are available in Additional File 2. Interestingly, relatively few proteins were found to be differentially expressed in these experiments. This finding is likely the result of a combination of factors, including our decision to make conservative choices in the decision tree, as well as the fact that the observed fold-changes are small for most of the differentially expressed proteins.

**Table 1:** Protein expression changes induced by TGF-β (24 hour treatment) in BRI-JM01 cells, identified by WGA affinity workflow.

	**BR1**		**BR2**		**BR3**	
**Swiss-Prot**	**# pept**	**ratio^1 ^± SD**	**# pept**	**ratio ± SD**	**# pept**	**ratio ± SD**
***UP-REGULATED***						
Q06890**|CLUS_MOUSE Clusterin precursor**						
	9	4.21 ± 0.51	7	4.26 ± 0.17	3	4.25 ± 0.45
P11276**|FINC_MOUSE Fibronectin precursor (FN)**						
	7	1.85 ± 0.37	8	1.63 ± 0.69	3	1.92 ± 0.57
P11688**|ITA5_MOUSE Integrin alpha-5**						
	2	1.41 ± 0.21	2	1.97 ± 0.16	1	9.93 ± 1.33
NP_997551.1**| Acid phosphatase, prostate isoform 1 [NP_062781.2]**						
	1	1.40 ± 0.17	1	1.49 ± 0.21	0	--
O70309**|ITB5_MOUSE Integrin beta-5**						
	2	1.39 ± 0.14	2	0.99 ± 0.16	2	1.55 ± 0.10
NP_058085.1**| Heterogeneous nuclear ribonucleoprotein U**						
	2	1.36 ± 0.6	2	1.37 ± 0.27	0	--
P63260**|ACTG_MOUSE Actin, cytoplasmic 2 [P60710]**						
	8	1.34 ± 0.21	10	1.13 ± 0.08	6	1.62 ± 0.08
						
***DOWN-REGULATED***						
Q9QZM0**|UBQL2_MOUSE Ubiquilin-2**						
	1	0.91 ± 0.71	3	0.69 ± 0.08	1	0.64 ± 0.16
Q8R143**|PTTG_MOUSE Pituitary tumor-transforming gene 1**						
	2	0.80 ± 0.05	2	0.70 ± 0.04	0	--
P35951**|LDLR_MOUSE Low-density lipoprotein receptor precursor**						
	1	0.62 ± 0.11	1	0.71 ± 0.06	0	--
Q61739**|ITA6_MOUSE Integrin alpha-6**						
	13	0.57 ± 0.08	13	0.79 ± 0.04	4	0.87 ± 0.03
NP_598424.2**| Integrin beta 4**						
	15	0.56 ± 0.08	15	0.84 ± 0.05	3	0.98 ± 0.22
NP_064431.2**| tumor-associated calcium signal transducer 2**						
	2	0.51 ± 0.12	2	0.57 ± 0.05	0	--

^1^abundance in TGF-β/abundance in CTL

**Table 2:** Protein expression changes induced by TGF-β (24 hour treatment) in NMuMG cells, identified by WGA affinity workflow.

	**BR1**		**BR2**		**BR3**	
**Swiss-Prot**	**# pept**	**ratio^1 ^± SD**	**# pept**	**ratio ± SD**	**# pept**	**ratio ± SD**
***UP-REGULATED***						
P13595**|NCA11_MOUSE Neural cell adhesion molecule 1, 180 kD [P13594]**						
	2	2.29 ± 0.64	2	3.02 ± 0.3	3	2.82 ± 0.61
P11276**|FINC_MOUSE Fibronectin precursor (FN)**						
	1	2.02 ± 0.79	1	2.37 ± 0.54	1	2.68 ± 0.45
P11688**|ITA5_MOUSE Integrin alpha-5**						
	2	1.75 ± 0.32	2	5.29 ± 0.8	2	1.55 ± 0.44
P37889**|FBLN2_MOUSE Fibulin-2 precursor**						
	0	--	1	2.31 ± 0.3	1	3.18 ± 0.23
						
***DOWN-REGULATED***						
Q9QZM0**|UBQL2_MOUSE Ubiquilin-2**						
	6	0.65 ± 0.1	3	0.64 ± 0.13	5	0.47 ± 0.09
Q62470**|ITA3_MOUSE Integrin alpha-3**						
	2	0.72 ± 0.1	3	0.61 ± 0.04	5	0.76 ± 0.08
Q9Z0K8**|VNN1_MOUSE Pantetheinase**						
	3	0.26 ± 0.02	3	0.53 ± 0.05	3	0.91 ± 0.31
O08573**|LEG9_MOUSE Galectin-9**						
	1	0.39 ± 0.08	1	0.46 ± 0.06	1	0.86 ± 0.07
NP_038820.1**| alpha-N-acetylglucosaminidase**						
	1	0.32 ± 0.06	1	0.80 ± 0.11	1	0.40 ± 0.12
P18572**|BASI_MOUSE Basigin**						
	1	0.41 ± 0.11	1	0.61 ± 0.07	2	0.81 ± 0.1

^1^abundance in TGF-β/abundance in CTL

**Figure 6:**
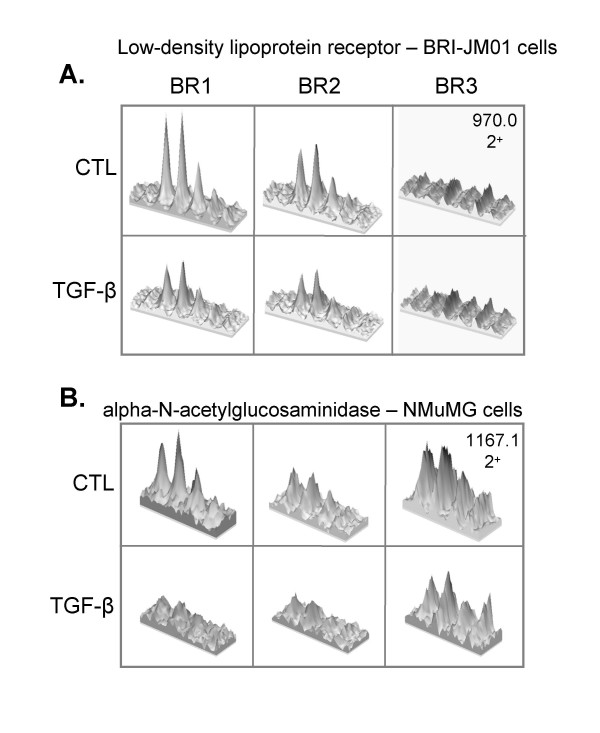
**Representative LC-MS quantification data**. Three-dimensional extracted ion chromatograms, produced by MSight [30] are shown for two peptides that are down-regulated upon the induction of EMT by TGF-β. (A) In BRI-JM01 cells, a doubly-charged peptide from the low-density lipoprotein receptor was identified with m/z of 970.0 m/z, representing the peptide sequence NIYWTDSVPGSVSVADTK. LC-MS data from the first 2 biological repetitions show consistently lower MS signal for this peptide upon TGF-β treatment. However, the peptide was not found in the third biological repeat. (B) In NMuMG cells, a doubly-charged peptide from alpha-N-acetylglucosaminidase was identified with m/z 1167.1, representing the sequence ALADESGLDTYSLSGGGGVPVLVR. The MS signal corresponding to this peptide is consistently lower after induction of EMT by TGF-β than in control cells.

Figure 6 shows representative LC-MS quantitative data for two down-regulated proteins that were identified by a single peptide: low-density lipoprotein receptor (LDLR) in BRI-JM01 cells and α-N-acetylglucosaminidase in NMuMG cells. For each of these peptides, the MS signal is consistently lower in the TGF-β sample than the control sample in multiple biological repetitions. The LDLR peptide was not visible in the third biological repetition, possibly due to poorer chromatographic resolution for this repetition, relative to the others.

Approximately 70% of the proteins in Table 1 and Table 2 are known to be glycoproteins containing N-linked glycosylation sites. The remaining proteins consist of proteins that physically bind to glycoproteins, such as Galectin-9 and Ubiquilin 2, or abundant cytoskeletal proteins, such as actin, which may also form complexes with glycosylated proteins. Alternatively, the presence of these abundant proteins may also result from non-specific binding to the WGA resin.

To further validate the findings from this study, we used four different methods: comparison to microarray data, multiple reaction monitoring (MRM), western blotting, and immunofluorescence microscopy. First, we determined whether microarray experiments which had previously been performed on the BRI-JM01 cell system revealed up- or down-regulation of the mRNA transcripts that encode for the proteins in Table 1. In the microarray study, we evaluated the transcriptional changes in BRI-JM01 cells exposed for 0.5, 1, 2, 4, 6, 12, and 24 hrs to TGF-β1 using cDNA microarrays containing 15,264 sequence verified mouse ESTs (University Health Network Microarray Centre in Toronto; ). Data was normalized (Lowess algorithm) and 328 significantly modulated genes were identified (False Discovery Rate < 10%) with minimally a 1.3 fold-variation in at least one of the time points, using the SAM one class algorithm (GeneSpring, Agilent Technologies). We found that 4 of the proteins found to be differentially expressed in this WGA study showed analogous statistically-significant changes in mRNA transcript levels (Lenferink *et al.*, manuscript in preparation). These proteins include Clusterin, Fibronectin, Integrin α5, and Integrin α6, whose mRNA levels were modulated 6.5, 1.35, 3.74, and 0.52-fold, respectively.

**Figure 7:**
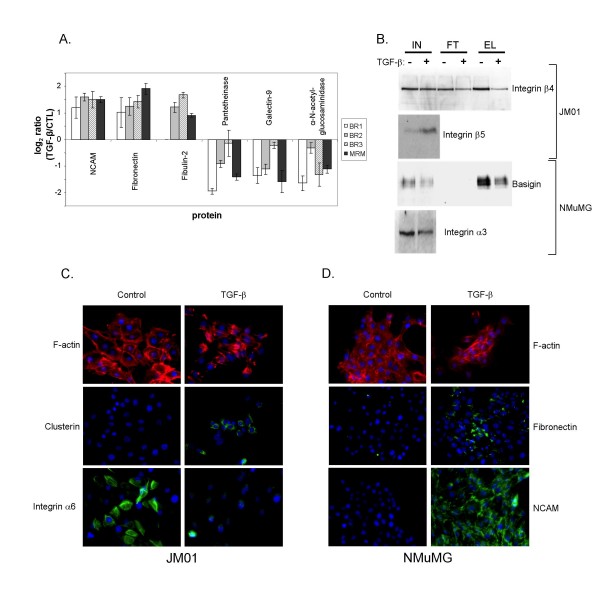
**Validation of TGF-β induced changes in protein expression levels identified by WGA affinity workflow**. (A) The ratio of peptide abundance identified by selectively monitoring three transitions from a peptide by multiple-reaction monitoring (MRM, shown as black bars) derived from each of six proteins is compared to the results from the original analysis of the first three biological repetitions (BR1, white bars; BR2, gray bars, and BR3, hashed bars). Protein ratios are shown as log2 of the ratio of abundance in TGF-β treated NMuMG cells relative to non-treated CTL cells. MRM results are as follows when presented as fold-change ± standard deviation, similar to those shown in Table 2: Neural cell adhesion molecule 1 (NCAM; 2.82 ± 0.22), Fibronectin (3.74 ± 0.51), Fibulin-2 (1.86 ± 0.10), Pantetheinase (0.38 ± 0.03), Galectin-9 (0.33 ± 0.10), and α-N acetylglucosaminidase (0.46 ± 0.04). (B) Western blot analysis of unfractionated cell lysate (IN), WGA flow-through (FT) and elution (EL) fractions of JM01 and NMuMG cells cultured in the absence (-) or presence (+) of TGF-β1 for 24 hrs. The protein levels of Integrin β4 (JM01), Basigin, and Integrin α3 all reduced upon treatment of the cells with TGF-β1, whereas those of Integrin β5 (JM01) increased. Using immunofluorescence microscopy (C, D) the occurrence of EMT in the JM01 and NMuMG was confirmed by the reorganization of the F-actin filaments (red, top panels), and is accompanied by the TGF-β induced upregulation of Clusterin and down-regulation of Integrin α6 in the JM01 cell line (C), and the up-regulation of Fibronectin and NCAM in the NMuMG cell line (D).

Since microarray data was not available in house for the conditions used in this study with the NMuMG cells, we chose to validate some of the proteins in Table 2 using a mass spectrometry-based multiple reaction monitoring (MRM) approach. For these experiments, glycoproteins from CTL and TGF-β-treated NMuMG cells from a fourth biological repetition were enriched using WGA and three fragment-ions from peptides derived from Neural cell adhesion molecule (NCAM), Fibronectin, Fibulin-2, Panthetheinase, Galectin-9, and alpha-N-glycosaminidase were selectively monitored. In each case, the MRM analysis agreed very well with the LC-MS quantification from the first 3 biological repetitions, as shown in Figure 7A. In this figure, log_2 _of the ratio of peptide abundance in TGF-β-treated NMuMG cells, relative to CTL cells, is plotted. Thus, peptides with a positive log_2 _ratio are up-regulated and peptides with a negative log_2 _ratio are down-regulated. Attempts to monitor these same ions in the unfractionated NMuMG cell lysate were not successful due to high background noise levels that swamped out the signal from the specific transition being monitored. The high noise level is likely due to the nature of the ion trap mass selection, which first fills the trap with all ions being produced before selectively isolating the ion of interest. Thus, unlike a triple quadrupole instrument, the sensitivity is limited by both the complexity of the total ions being produced and the number of ions that can be contained in the trap. Nonetheless, we found that MRM in an ion trap instrument worked well to confirm findings in our WGA elution fractions.

Four proteins were validated by western blot analysis. In JM01 cells, the protein levels of Integrin β4 were confirmed to be down-regulated whereas those of Integrin β5 were up-regulated. In NMuMG cells, both Basigin and Integrin α3, were confirmed to be down-regulated (Figure 7B). Importantly, Integrin β5, Basigin, and Integrin α3 are modulated in the unfractionated cellular lysate ('input'). This finding demonstrates that the expression level of these proteins is regulated upon the induction of EMT. In contrast, Integrin β4 appears to be selectively down-regulated in the lectin-enriched fraction, suggestive of a modification in glycan structure that affects the affinity of Integrin β4 binding to WGA. Without this validation data from the cell lysate, we can not definitively distinguish between changes in protein expression levels and changes in glycosylation status. A change in a protein's binding affinity for WGA could change if the glycosylation of a protein is modified, either by a change in glycan site occupancy or by a change in glycan structure. Glycosylation modifications such as these would result in a change in abundance in the WGA elution fraction, but would not affect the total protein expression level in the cell lysate. We believe that most of the proteins found to be regulated using this experimental approach are observed due to overall changes in protein expression levels, rather than changes in glycosylation status, since we were able to validate the majority by microarray or western blot analysis as having their expression level changed. However, we can not eliminate modifications in glycosylation for some of the proteins listed in Tables 1 and 2.

Immunofluorescence microscopy was also used to validate expression changes in several of the proteins identified by the WGA workflow. Since the reorganization of filamentous actin (F-actin) is one of the hallmarks of a TGF-β induced EMT, we visualized F-actin using rhodamine-conjugated phalloidin. Figure 7C (JM01, top panel) and 7D (NMuMG, top panel) show the rearrangement of F-actin in both the JM01 and NMuMG cells upon the treatment with TGF-β (24 hrs), confirming that EMT has occurred. We then demonstrated increased Clusterin and Integrin β5 protein levels and decreased levels of Integrin α6 in JM01 cells (Figure 7C) and the up-regulation of Fibronectin and NCAM and down-regulation of Integrin α3 in NMuMG cells (Figure 7D) upon treatment with TGF-β.

A largely distinct list of protein changes was found in the 2DE study and in the lectin study. With the exception of Integrin α5, there was no overlap in the proteins identified. As is becoming increasingly apparent, use of a different workflow for sample preparation and analysis for proteomic studies tends to identify different subsets of proteins. Thus, there is a clear advantage to using complementary techniques to identify protein expression changes.

Both the 2DE and lectin experiments suggest that the abundance of the vast majority of proteins is not affected by TGF-β treatment. As a whole, TGF-β-induced changes in protein expression were relatively subtle in these cell systems. These subtle changes are consistent with microarray studies on the BRI-JM01 cell line. In this study, only 8 out of 328 regulated genes had their transcripts up-regulated by more than 2-fold (Lenferink *et al.*, manuscript in preparation). It is also consistent with a proteomic study that used iTRAQ to quantify protein changes in the TGF-β-induced EMT in A549 human lung cancer cells [10]. In this study, only 2 of the 51 regulated proteins identified had a greater than 2-fold change. It seems somewhat surprising that the dramatic phenotypic change associated with EMT is able to occur in the presence of only subtle changes in protein abundance. These findings point to the possibility that changes in post-translational modifications, such as glycosylation or phosphorylation, may play a more important role in mediating these phenotypic changes. In fact, glycosylation has been shown to affect cell migration, tumor invasion, and many other cellular processes [32]. The lectin used for enrichment in these experiments, WGA, has a relatively broad binding specificity for N-linked glycans and is therefore not particularly well-suited for teasing out modifications to glycan structure. In experiments similar to those presented here, it would be informative to use a variety of lectins that recognize more specific glycan structures as an extension of our experimental paradigm. In particular, it would prove interesting to focus on lectins which recognize glycan structures that are known to be altered in cancer.

### Biological implications of selected protein expression changes that occur during TGF-β induced EMT

Tumors are known to be highly heterogenous at the molecular level, even between tumors with the same histopathological classification. Accordingly, different cancer cells may utilize distinct molecular mechanisms to achieve similar phenotypic changes characteristic of EMT. Thus, the identification of EMT-associated changes in protein expression that are common among different cell lines should focus on the events that play more central roles in the regulation of tumor cell invasion and metastasis. Despite similarities in cell type and phenotypic response to TGF-β, only three proteins were found to be regulated in a similar manner in both BRI-JM01 and NMuMG cells. This may reflect some biological variation in the mechanisms used to induce EMT by TGF-β in the two cell lines. However, the dissimilarity between the results may also be due to technical reasons. Many proteins that were quantified in one cell line were not quantified in the other. Also, the conservative choices that we used to define differentially expressed proteins will mean that some differentially-expressed proteins are likely to be missed (were false negatives). This is especially true if fewer peptides belonging to that protein were identified, as would result from differences in the abundance or post-translational modification of a particular protein between the two cell lines. As in the vast majority of proteomic studies, it is possible to list what was observed as changing in these experiments, but more difficult to say what was missed. Thus, additional proteins may be similarly regulated in both cell lines, even though they were not found in these experiments.

The three protein changes that were identified in both the BRI-JM01 and NMuMG cell lines are the up-regulation of Fibronectin, the up-regulation of Integrin α5, and the down-regulation of Ubiquilin-2. Fibronectin is a known marker for mesenchymal cells, so its up-regulation during epithelial-to-mesenchymal transition is expected [33]. The expression of Integrin α5 has previously been shown to increase during the TGF-β-induced EMT in Ras-transformed EpH4 mammary epithelial cells. Furthermore, blocking the function of Integrin α5 in these cells with an antibody was shown to prevent the induction of EMT upon TGF-β treatment, demonstrating a key role for this protein in EMT progression [34]. Unlike Fibronectin and Integrin α5, Ubiquilin-2 does not have any known ties to EMT or cancer. Little is known about Ubiquilin-2, but it is thought to play a role in regulating protein degradation by physically linking the ubiquitination machinery with the proteasome [35].

In BRI-JM01 cells, the induction of EMT led to protein level expression changes in several members of the Integrin family. Integrins are single-pass type I membrane proteins that function as heteromeric dimers, consisting of an α and β subunit, that play an important role in the regulation of tumor cell invasion by linking the extracellular matrix to the cytoskeleton [36,37]. Integrins have previously been shown be involved in the EMT process during murine lens development [38], trophoblast differentiation [39], prostate cancer cell migration [40], and tumor stem cell development [41]. In BRI-JM01 cells, TGF-β up-regulated the expression of Integrin α5, Integrin α2, and Integrin β5. To date, one N-linked glycosylation site has been characterized on each of the α chains [42,43] and many reports have demonstrated that *N*-glycosylation of Integrins affects their biological activity, especially the binding towards ECM macromolecules [44,45]. Interestingly, the Integrin heterodimer α5/β1 is associated with the deposition of and adhesion to Fibronectin, a characteristic of invasive breast carcinoma cells [34]. Thus, the up-regulation of these Integrin subunits may directly contribute to the increased expression of Fibronectin seen in these experiments. In contrast, TGF-β lowered the expression of both Integrin α6 and Integrin β4. Together, these integrins form one of the major cellular receptors for Laminin [46]. Interestingly, the level of down-regulation of Integrin α6 and Integrin β4 correlated well with each other in the three biological repeats analyzed possibly indicating a common mechanism regulating their expression. Knock-out mouse studies have shown that the Integrin α6/β4 heterodimer maintains the structural integrity of the epidermis by linking epithelial cells to the basement membrane [47,48]. Thus, lowering expression of α6/β4 may be partially responsible for the lowered cell-cell adhesion seen after EMT. The role of α6/β4 in cancer is more complex – in many cases this integrin is up-regulated carcinoma cells, where it may contribute to invasion and motility by regulating intracellular signaling events [49].

One of the largest protein expression changes we observed in the BRI-JM01 cell line is that of Clusterin, a 70–80 kDa glycoprotein that has been implicated in several mechanisms that drive carcinogenesis and tumor progression [50]. Clusterin is expressed in a variety of cancers and is associated with broad-spectrum treatment resistance. Custirsen (OGX-011), a 2'-methoxyethyl modified phosphorothioate antisense oligonucleotide that is complementary to clusterin mRNA, is currently in clinical trails.

Despite similarities in the phenotypic changes induced by TGF-β, most protein changes in NMuMG cells appear to be largely distinct from those in BRI-JM01 cells. One of the largest protein expression changes in NMuMG cells is the up-regulation of Neural Cell Adhesion Molecule 1 (NCAM-1). Although NCAM-1 is known to be overexpressed in many brain cancers, small cell lung cancer, and multiple myeloma, is has not generally been discussed as a marker for breast cancer [51]. Interestingly, many cancers are known to overexpress a different cell adhesion molecule, called L1, which has been shown to regulate EMT-like events in transformed MCF7 breast carcinoma cells [52]. Intriguingly, NCAM has been shown to enhance the homophilic interaction between L1 molecules, suggesting that these 2 cell adhesion molecules may work together to regulate cell-cell interactions [53]. Recently, the up-regulation of NCAM was shown to be the direct result of the loss of adherens junctions. The clustering of NCAM with p59^Fyn ^in lipid rafts resulted in the phosphorylation of FAK and the assembly of focal adhesions, both of which are required for EMT and cell motility [54].

The WGA workflow also revealed that Fibulin-2 is up-regulated after EMT in NMuMG cells. Fibulin-2 is a member of a family of secreted glycoproteins that interact with extracellular matrix proteins to form intramolecular bridges [55]. Fibulin proteins have been associated with both tumor progression and tumor suppression, a paradox that may be the result of alternative splicing events, which lead to Fibulin variants with different functional roles [55]. Recently, the loss of Fibulin-2 has been shown to be associated with breast cancer progression [56]. Conversely, a recent proteomics-based study identified Fibulin-2 as a marker for breast cancer in a conditional HER2/Neu-driven mouse model [57]. The authors of this study were further able to show that Fibulin-2 has promise, not only as a tissue biomarker of cancer, but also as a plasma biomarker.

Although many of the proteins identified as associated with EMT in these experiments have been previously linked to cancer, several have not previously been associated with cancer progression or the EMT process. For example, using 2DE, we found that Tropomodulin 3 is overexpressed upon TGF-β treatment of JM01 cells. Tropomodulin 3 (Tmod 3) is a widely expressed protein that caps the pointed ends of actin filaments, as well as binding and sequestering actin monomers [58]. Through these interactions, Tmod 3 regulates dynamic actin networks, such as those in the lamellipodia of motile endothelial cells. Interestingly, Tmod 3 is generally thought to act as a negative regulator of cell migration [59]. The fact that TGF-β treatment increases Tmod 3 expression while also increasing cell motility in JM01 cells suggests that cell motility may be regulated by a different mechanism during the EMT. Another protein that has not been previously linked to cancer progression or EMT is alpha-N-acetylglycosaminidase (Naglu), which is down-regulated in NMuMG cells upon the induction of EMT. Naglu is a lysosomal enzyme required for the degradation of the polysaccharide, heparan sulfate. Mutations in the gene for Naglu are the cause of mucopolysaccharidosis IIIB, a lysosomal storage disease characterized by buildup of glycosaminoglycan[60]. Interestingly, the degradation of extracellular heparan sulfate by another enzyme, heparanase, has been strongly linked to breast cancer invasion and progression[61]. Thus, the down-regulation of Naglu may also contribute to cancer progression through the regulation of heparan sulfate metabolism inside the cell.

## Conclusion

Using a combination of 2DE and a lectin affinity-MS quantification workflow, we have identified 13 proteins that are up-regulated and 11 proteins that are down-regulated in two mouse mammary epithelial cell lines, BRI-JM01 and NMuMG, upon the induction of EMT by TGF-β. Tumors are known to be highly heterogenous, even between tumors with the same histopathological classification. By studying the EMT process in two independently isolated cell lines, we aimed at focusing on proteins that are centrally involved in mediating cancer cell motility and invasiveness. Surprisingly, despite strong similarities in phenotypic response to TGF-β (i.e. the acquisition of a fibroblast-like morphology), BRI-JM01 and NMuMG cells undergo largely distinct protein expression changes upon EMT induction. This lack of overlap is likely due in part to the conservative cutoff values used in these experiments, as well as the limitations of analyzing only the subset of proteins that bind to WGA. It is also possible that the two cell lines use somewhat different mechanisms to achieve an EMT transition. This finding highlights the need for analyzing multiple cell lines and systems to find common protein changes in order to identify more useful diagnostic markers and therapeutic targets for cancer treatment. To conclude, the study described here demonstrates the use of glycoproteomics to define potential glycoprotein biomarkers and therapeutic targets. The discovery and subsequent validation of such glycoproteins will allow for the development of drug candidates that neutralize their function, as well as diagnostic tools that can be used to monitor disease progression, recurrence, and response to treatment.

## Competing interests

The authors declare that they have no competing interests.

## Authors' contributions

JJH conceived, designed, and performed the lectin affinity and mass spectrometry work, performed the statistical analysis, participated in the biological discussion, and drafted the manuscript. TLT performed the two-dimensional gel studies and participated in the western blot analysis. CC performed the immunofluorescence microscopy. MO initiated the TGF-beta work and participated in the coordination of the study. JK conceived the focus on glycoproteomics and participated in the coordination of the study. AEL conceived, designed, and performed western blot analysis and cell culture, designed the immunofluorescence validation, and participated in the biological discussion. All authors edited the manuscript and approved the final version.

## Supplementary Material

Additional file 1 **Supplementary Figures 1 and 2**. As described in the text, Supplementary Figure 1 and Supplementary Figure 2 and their corresponding legends are provided in this file. Click here for file

Additional file 2 **Peptide Details.** The data provided contains peptide level quantification and database search results for the peptides derived from the proteins listed in Table 1 and Table 2. Click here for file

## References

[B1] Jakowlew SB (2006). Transforming growth factor-beta in cancer and metastasis. Cancer Metastasis Rev.

[B2] Guarino M, Rubino B, Ballabio G (2007). The role of epithelial-mesenchymal transition in cancer pathology. Pathology.

[B3] Larue L, Bellacosa A (2005). Epithelial-mesenchymal transition in development and cancer: role of phosphatidylinositol 3' kinase/AKT pathways. Oncogene.

[B4] Kang Y, Massague J (2004). Epithelial-mesenchymal transitions: twist in development and metastasis. Cell.

[B5] Thiery JP, Morgan M (2004). Breast cancer progression with a Twist. Nat Med.

[B6] Trainor PA, Melton KR, Manzanares M (2003). Origins and plasticity of neural crest cells and their roles in jaw and craniofacial evolution. Int J Dev Biol.

[B7] Lenferink AE, Magoon J, Cantin C, O'Connor-McCourt MD (2004). Investigation of three new mouse mammary tumor cell lines as models for transforming growth factor (TGF)-beta and Neu pathway signaling studies: identification of a novel model for TGF-beta-induced epithelial-to-mesenchymal transition. Breast Cancer Res.

[B8] Miettinen PJ, Ebner R, Lopez AR, Derynck R (1994). TGF-beta induced transdifferentiation of mammary epithelial cells to mesenchymal cells: involvement of type I receptors. J Cell Biol.

[B9] Demir AY, Demol H, Puype M, de Goeij AF, Dunselman GA, Herrler A, Evers JL, Vandekerckhove J, Groothuis PG (2004). Proteome analysis of human mesothelial cells during epithelial to mesenchymal transitions induced by shed menstrual effluent. Proteomics.

[B10] Keshamouni VG, Michailidis G, Grasso CS, Anthwal S, Strahler JR, Walker A, Arenberg DA, Reddy RC, Akulapalli S, Thannickal VJ (2006). Differential protein expression profiling by iTRAQ-2DLC-MS/MS of lung cancer cells undergoing epithelial-mesenchymal transition reveals a migratory/invasive phenotype. J Proteome Res.

[B11] Moreira JM, Gromov P, Celis JE (2004). Expression of the tumor suppressor protein 14-3-3 sigma is down-regulated in invasive transitional cell carcinomas of the urinary bladder undergoing epithelial-to-mesenchymal transition. Mol Cell Proteomics.

[B12] Willipinski-Stapelfeldt B, Riethdorf S, Assmann V, Woelfle U, Rau T, Sauter G, Heukeshoven J, Pantel K (2005). Changes in cytoskeletal protein composition indicative of an epithelial-mesenchymal transition in human micrometastatic and primary breast carcinoma cells. Clin Cancer Res.

[B13] Dwek MV, Ross HA, Leathem AJ (2001). Proteome and glycosylation mapping identifies post-translational modifications associated with aggressive breast cancer. Proteomics.

[B14] Ono M, Hakomori S (2004). Glycosylation defining cancer cell motility and invasiveness. Glycoconj J.

[B15] Cho WC (2007). Contribution of oncoproteomics to cancer biomarker discovery. Mol Cancer.

[B16] Macher BA, Yen TY (2007). Proteins at membrane surfaces-a review of approaches. Mol Biosyst.

[B17] Drake RR, Schwegler EE, Malik G, Diaz J, Block T, Mehta A, Semmes OJ (2006). Lectin capture strategies combined with mass spectrometry for the discovery of serum glycoprotein biomarkers. Mol Cell Proteomics.

[B18] Kaji H, Yamauchi Y, Takahashi N, Isobe T (2006). Mass spectrometric identification of N-linked glycopeptides using lectin-mediated affinity capture and glycosylation site-specific stable isotope tagging. Nat Protoc.

[B19] Plavina T, Wakshull E, Hancock WS, Hincapie M (2007). Combination of abundant protein depletion and multi-lectin affinity chromatography (M-LAC) for plasma protein biomarker discovery. J Proteome Res.

[B20] Ueda K, Katagiri T, Shimada T, Irie S, Sato TA, Nakamura Y, Daigo Y (2007). Comparative profiling of serum glycoproteome by sequential purification of glycoproteins and 2-nitrobenzensulfenyl (NBS) stable isotope labeling: a new approach for the novel biomarker discovery for cancer. J Proteome Res.

[B21] Reardon DA, Akabani G, Coleman RE, Friedman AH, Friedman HS, Herndon JE, McLendon RE, Pegram CN, Provenzale JM, Quinn JA (2006). Salvage radioimmunotherapy with murine iodine-131-labeled antitenascin monoclonal antibody 81C6 for patients with recurrent primary and metastatic malignant brain tumors: phase II study results. J Clin Oncol.

[B22] Dai Z, Fan J, Liu Y, Zhou J, Bai D, Tan C, Guo K, Zhang Y, Zhao Y, Yang P (2007). Identification and analysis of alpha1,6-fucosylated proteins in human normal liver tissues by a target glycoproteomic approach. Electrophoresis.

[B23] Saint-Guirons J, Zeqiraj E, Schumacher U, Greenwell P, Dwek M (2007). Proteome analysis of metastatic colorectal cancer cells recognized by the lectin Helix pomatia agglutinin (HPA). Proteomics.

[B24] Brockhausen I, Schutzbach J, Kuhns W (1998). Glycoproteins and their relationship to human disease. Acta Anat (Basel).

[B25] Kanninen K, Goldsteins G, Auriola S, Alafuzoff I, Koistinaho J (2004). Glycosylation changes in Alzheimer's disease as revealed by a proteomic approach. Neurosci Lett.

[B26] Lu X, Zhu H (2005). Tube-gel digestion: a novel proteomic approach for high throughput analysis of membrane proteins. Mol Cell Proteomics.

[B27] Haqqani AS, Kelly JF, Stanimirovic DB, Starkey M, Elaswarapu R (2008). Quantitative protein profiling by mass spectrometry using label-free proteomics. Methods in Molecular Biology: Genomics Protocols.

[B28] Altschul SF, Madden TL, Schaffer AA, Zhang J, Zhang Z, Miller W, Lipman DJ (1997). Gapped BLAST and PSI-BLAST: a new generation of protein database search programs. Nucleic Acids Res.

[B29] Schaffer AA, Aravind L, Madden TL, Shavirin S, Spouge JL, Wolf YI, Koonin EV, Altschul SF (2001). Improving the accuracy of PSI-BLAST protein database searches with composition-based statistics and other refinements. Nucleic Acids Res.

[B30] Palagi PM, Walther D, Quadroni M, Catherinet S, Burgess J, Zimmermann-Ivol CG, Sanchez JC, Binz PA, Hochstrasser DF, Appel RD (2005). MSight: an image analysis software for liquid chromatography-mass spectrometry. Proteomics.

[B31] Pardali K, Moustakas A (2007). Actions of TGF-beta as tumor suppressor and pro-metastatic factor in human cancer. Biochim Biophys Acta.

[B32] Zhao YY, Takahashi M, Gu JG, Miyoshi E, Matsumoto A, Kitazume S, Taniguchi N (2008). Functional roles of N-glycans in cell signaling and cell adhesion in cancer. Cancer Sci.

[B33] Thiery JP, Sleeman JP (2006). Complex networks orchestrate epithelial-mesenchymal transitions. Nat Rev Mol Cell Biol.

[B34] Maschler S, Wirl G, Spring H, Bredow DV, Sordat I, Beug H, Reichmann E (2005). Tumor cell invasiveness correlates with changes in integrin expression and localization. Oncogene.

[B35] Kleijnen MF, Shih AH, Zhou P, Kumar S, Soccio RE, Kedersha NL, Gill G, Howley PM (2000). The hPLIC proteins may provide a link between the ubiquitination machinery and the proteasome. Mol Cell.

[B36] Gu J, Taniguchi N (2004). Regulation of integrin functions by N-glycans. Glycoconj J.

[B37] Jin H, Varner J (2004). Integrins: roles in cancer development and as treatment targets. Br J Cancer.

[B38] Barbour W, Saika S, Miyamoto T, Ohkawa K, Utsunomiya H, Ohnishi Y (2004). Expression patterns of beta1-related alpha integrin subunits in murine lens during embryonic development and wound healing. Curr Eye Res.

[B39] Vicovac L, Aplin JD (1996). Epithelial-mesenchymal transition during trophoblast differentiation. Acta Anat (Basel).

[B40] King TE, Pawar SC, Majuta L, Sroka IC, Wynn D, Demetriou MC, Nagle RB, Porreca F, Cress AE (2008). The role of alpha 6 integrin in prostate cancer migration and bone pain in a novel xenograft model. PLoS ONE.

[B41] Pontier SM, Muller WJ (2008). Integrins in breast cancer dormancy. Apmis.

[B42] Lewandrowski U, Moebius J, Walter U, Sickmann A (2006). Elucidation of N-glycosylation sites on human platelet proteins: a glycoproteomic approach. Mol Cell Proteomics.

[B43] Zhang H, Li XJ, Martin DB, Aebersold R (2003). Identification and quantification of N-linked glycoproteins using hydrazide chemistry, stable isotope labeling and mass spectrometry. Nat Biotechnol.

[B44] Isaji T, Sato Y, Zhao Y, Miyoshi E, Wada Y, Taniguchi N, Gu J (2006). N-glycosylation of the beta-propeller domain of the integrin alpha5 subunit is essential for alpha5beta1 heterodimerization, expression on the cell surface, and its biological function. J Biol Chem.

[B45] Zhao Y, Sato Y, Isaji T, Fukuda T, Matsumoto A, Miyoshi E, Gu J, Taniguchi N (2008). Branched N-glycans regulate the biological functions of integrins and cadherins. Febs J.

[B46] Mercurio AM, Rabinovitz I, Shaw LM (2001). The alpha 6 beta 4 integrin and epithelial cell migration. Curr Opin Cell Biol.

[B47] Dowling J, Yu QC, Fuchs E (1996). Beta4 integrin is required for hemidesmosome formation, cell adhesion and cell survival. J Cell Biol.

[B48] Neut R van der, Krimpenfort P, Calafat J, Niessen CM, Sonnenberg A (1996). Epithelial detachment due to absence of hemidesmosomes in integrin beta 4 null mice. Nat Genet.

[B49] Mercurio AM, Bachelder RE, Chung J, O'Connor KL, Rabinovitz I, Shaw LM, Tani T (2001). Integrin laminin receptors and breast carcinoma progression. J Mammary Gland Biol Neoplasia.

[B50] Shannan B, Seifert M, Leskov K, Willis J, Boothman D, Tilgen W, Reichrath J (2006). Challenge and promise: roles for clusterin in pathogenesis, progression and therapy of cancer. Cell Death Differ.

[B51] Jensen M, Berthold F (2007). Targeting the neural cell adhesion molecule in cancer. Cancer Lett.

[B52] Shtutman M, Levina E, Ohouo P, Baig M, Roninson IB (2006). Cell adhesion molecule L1 disrupts E-cadherin-containing adherens junctions and increases scattering and motility of MCF7 breast carcinoma cells. Cancer Res.

[B53] Kadmon G, Kowitz A, Altevogt P, Schachner M (1990). The neural cell adhesion molecule N-CAM enhances L1-dependent cell-cell interactions. J Cell Biol.

[B54] Lehembre F, Yilmaz M, Wicki A, Schomber T, Strittmatter K, Ziegler D, Kren A, Went P, Derksen PW, Berns A (2008). NCAM-induced focal adhesion assembly: a functional switch upon loss of E-cadherin. Embo J.

[B55] Gallagher WM, Currid CA, Whelan LC (2005). Fibulins and cancer: friend or foe?. Trends Mol Med.

[B56] Yi CH, Smith DJ, West WW, Hollingsworth MA (2007). Loss of fibulin-2 expression is associated with breast cancer progression. Am J Pathol.

[B57] Whiteaker JR, Zhang H, Zhao L, Wang P, Kelly-Spratt KS, Ivey RG, Piening BD, Feng LC, Kasarda E, Gurley KE (2007). Integrated pipeline for mass spectrometry-based discovery and confirmation of biomarkers demonstrated in a mouse model of breast cancer. J Proteome Res.

[B58] Fischer RS, Yarmola EG, Weber KL, Speicher KD, Speicher DW, Bubb MR, Fowler VM (2006). Tropomodulin 3 binds to actin monomers. J Biol Chem.

[B59] Fischer RS, Fritz-Six KL, Fowler VM (2003). Pointed-end capping by tropomodulin3 negatively regulates endothelial cell motility. J Cell Biol.

[B60] Yogalingam G, Hopwood JJ (2001). Molecular genetics of mucopolysaccharidosis type IIIA and IIIB: Diagnostic, clinical, and biological implications. Hum Mutat.

[B61] Gotte M, Yip GW (2006). Heparanase, hyaluronan, and CD44 in cancers: a breast carcinoma perspective. Cancer Res.

