# Supplementary material for: Glycoproteomic analysis of two mouse mammary cell lines during transforming growth factor (TGF)-β induced epithelial to mesenchymal transition
Source: Proteome Sci. 2009 Jan 8;7:2. doi: 10.1186/1477-5956-7-2 (PMC2651118; doi:10.1186/1477-5956-7-2)
Supplement: Additional file 1 — Supplementary Figures 1 and 2. As described in the text, Supplementary Figure 1 and Supplementary Figure 2 and their corresponding legends are provided in this file. [file 1477-5956-7-2-S1.pdf]

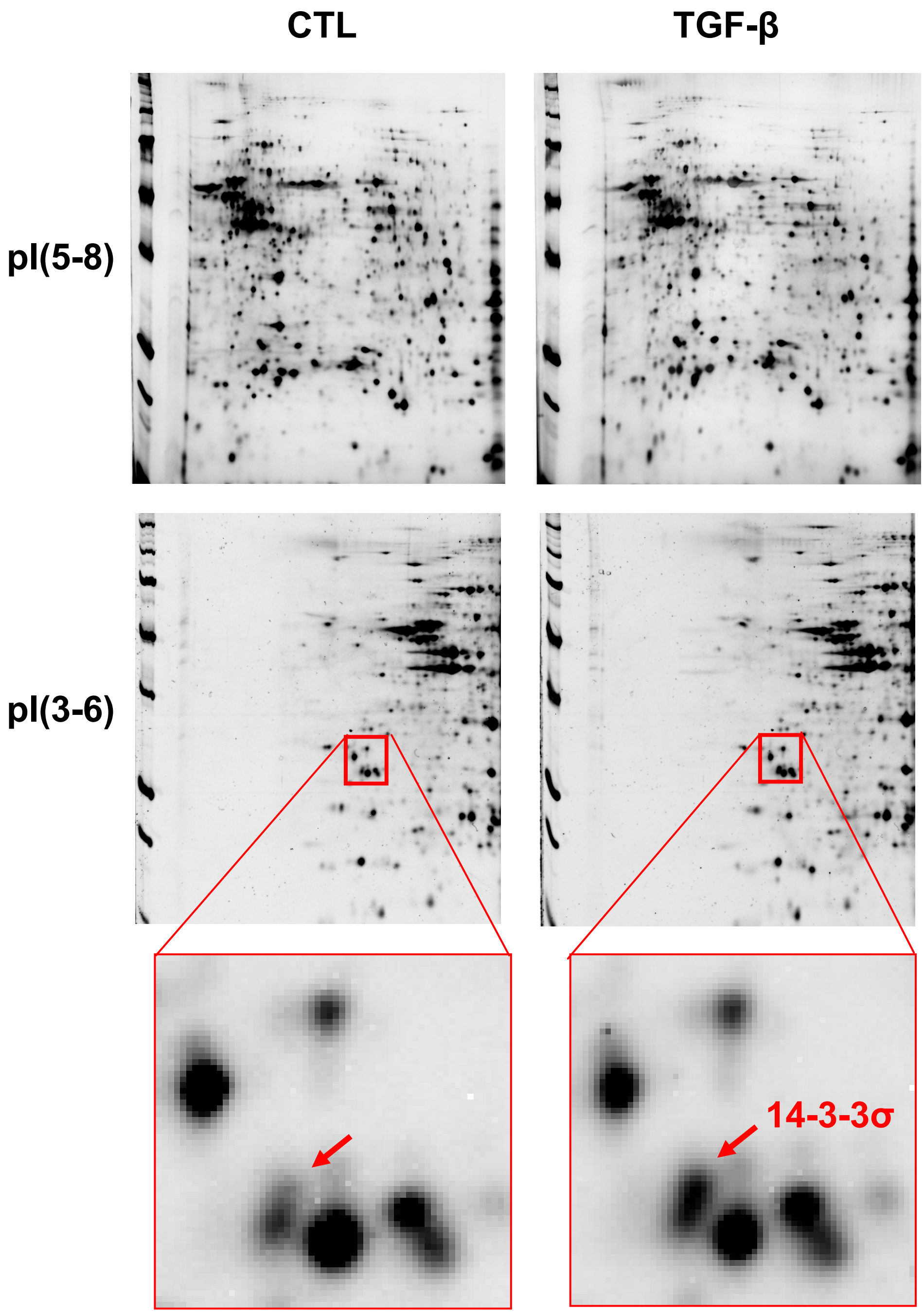

Supplementary Figure 1: Two-dimensional gels of hydrophobic-enriched fraction from BRI-JM01 control (CTL) and TGF-β treated cells separated in pI range 5-8 (top) and 3-6 (middle), showing high quality focusing and separation of protein spots. A zoomed region of the 3-6 gels that contains the up-regulated spot identified as 14-3-3σ is shown at bottom.

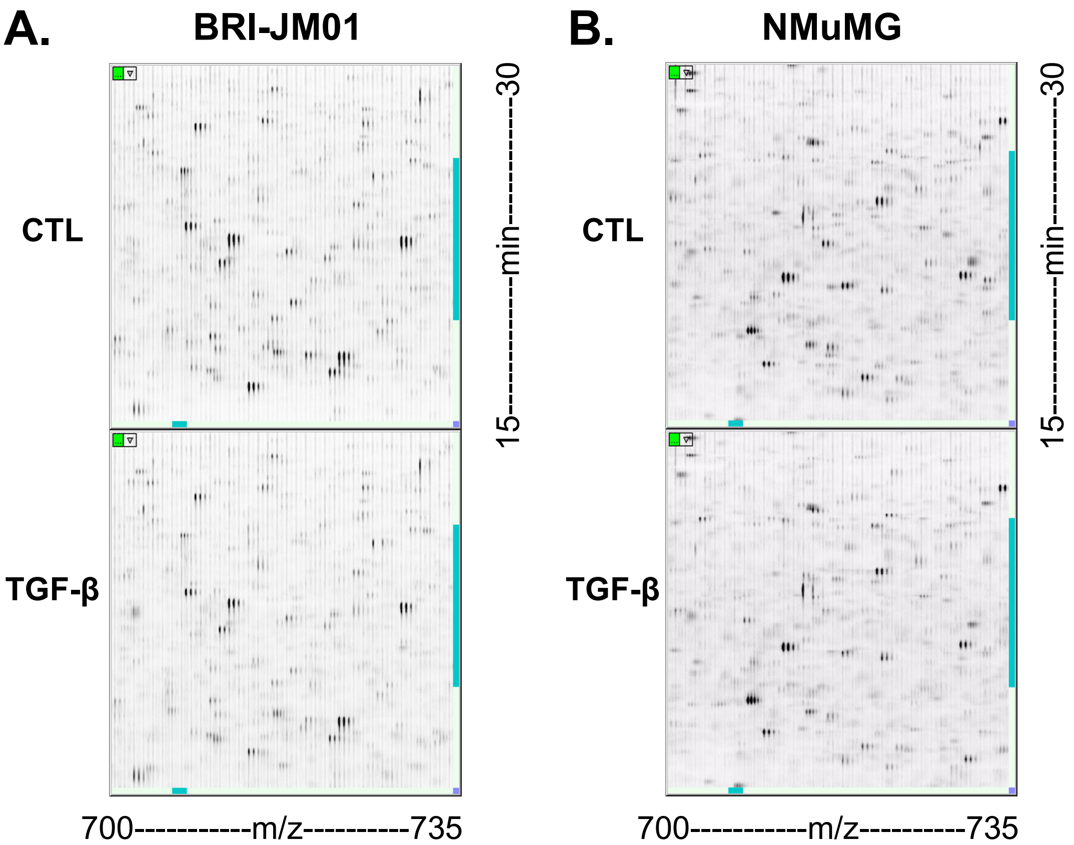

Supplementary Figure 2: Subsequent LC-MS analysis results in reproducible two-dimensional peptide maps, where each peptide is identified by m/z, z, and reverse phase retention time. Maps are shown above for representative LC-MS data from each WGA enriched sample from control and TGF-β treated BRI-JM01 (A) and NMuMG (B) cells. The maps were produced using the freely-available MSight software (http://www.expasy.org/).
